# Differences in brain morphology and working memory capacity across childhood

**DOI:** 10.1111/desc.12579

**Published:** 2017-07-26

**Authors:** Joe Bathelt, Susan E. Gathercole, Amy Johnson, Duncan E. Astle

**Affiliations:** ^1^ MRC Cognition and Brain Sciences Unit Cambridge UK

## Abstract

Working memory (WM) skills are closely associated with learning progress in key areas such as reading and mathematics across childhood. As yet, however, little is known about how the brain systems underpinning WM develop over this critical developmental period. The current study investigated whether and how structural brain correlates of components of the working memory system change over development. Verbal and visuospatial short‐term and working memory were assessed in 153 children between 5.58 and 15.92 years, and latent components of the working memory system were derived. Fractional anisotropy and cortical thickness maps were derived from T1‐weighted and diffusion‐weighted MRI and processed using eigenanatomy decomposition. There was a greater involvement of the corpus callosum and posterior temporal white matter in younger children for performance associated with the executive part of the working memory system. For older children, this was more closely linked with the thickness of the occipitotemporal cortex. These findings suggest that increasing specialization leads to shifts in the contribution of neural substrates over childhood, moving from an early dependence on a distributed system supported by long‐range connections to later reliance on specialized local circuitry. Our findings demonstrate that despite the component factor structure being stable across childhood, the underlying brain systems supporting working memory change. Taking the age of the child into account, and not just their overall score, is likely to be critical for understanding the nature of the limitations on their working memory capacity.

## RESEARCH HIGHLIGHTS

- Multiple measures of verbal and visuospatial short‐term and working memory enabling assessment of latent constructs of working memory, instead of using task‐specific scores.
- A large sample of children between 5.58 and 15.92 years who completed working memory assessments (*n* = 153), with a large subset who also completed T1‐weighted MRI (*n* = 122, age = 5.58–15.92y) and diffusion‐weighted MRI (*n* = 112, age = 5.58–15.92y).
- First study to investigate changes in the association between brain structures and working memory capacity across childhood and adolescence.

## 1 INTRODUCTION

Working memory is a limited capacity system for retaining and processing information over brief periods of time. It is closely linked with the acquisition of complex cognitive skills (Cowan, 2013) such as reading (Cain, Oakhill, & Bryant, 2004), mathematics (Dumontheil & Klingberg, 2011), and other academic subjects (Clair‐Thompson & Gathercole, 2006; Gathercole, Pickering, Knight, & Stegmann, 2003). Deficits in working memory have been identified across a range of neurodevelopmental disorders, including attention deficit hyperactivity disorder (Holmes et al., 2014; Martinussen, Hayden, Hogg‐Johnson, & Tannock, 2005), dyslexia (Smith‐Spark & Fisk, 2007), dyscalculia (Rotzer et al., 2009; Szucs, Devine, Soltesz, Nobes, & Gabriel, 2013), and language disorders (Archibald & Gathercole, 2006; Gathercole & Baddeley, 1989; Montgomery, 2000; Weismer, Evans, & Hesketh, 1999).

Working memory develops gradually through early and middle childhood (Gathercole, Pickering, Ambridge, & Wearing, 2004; Huizinga, Dolan, & van der Molen, 2006; Siegel & Ryan, 1988). It is assumed that this development reflects the maturation of the brain system supporting this skill in adulthood (Tamnes et al., 2013). However, understanding the mechanism of working memory development in childhood necessitates a neuropsychological account that incorporates developmental change. Currently, we have no detailed understanding of how age‐related changes in brain organization support specific developmental improvements in working memory. The purpose of this study is to take steps towards redressing this.

### 1.1 Working memory and its development

There are many theoretical accounts of working memory. The influential multicomponent model of working memory advanced by Baddeley and Hitch (Baddeley & Hitch, 1974) consists of three subcomponents: two domain‐specific stores and a central executive. The stores are specialized for the retention of material in either phonological (Baddeley, 1987) or visuospatial format (Baddeley & Lieberman, 1980; Logie, 1986). The central executive is a system responsible for a range of regulatory functions, including attention, the control of action, and problem solving (Baddeley, 1996).

There have been many refinements of the original model (Baddeley, 2000, 2003, 2012; Burgess & Hitch, 1996), and several new accounts. Some of these focus on specific mechanisms within working memory. For instance, Engle and colleagues propose inhibitory processes that protect activated memory traces from disruption (Engle, 2002; Kane, Conway, Hambrick, & Engle, 2007). Other models integrate short‐term memory with long‐term memory, suggesting that working memory represents long‐term memory in an activated state (Cowan, 1988, 1999; Oberauer, 2002), and activation is guided by an attentional mechanism. Other theorists have extended the scope of WM to encompass other processes that include updating (Ecker, Lewandowsky, Oberauer, & Chee, 2010; Schmiedek, Lövdén, & Lindenberger, 2014; Shelton, Elliott, Matthews, Hill, & Gouvier, 2010), set shifting and relational binding (Oberauer, Lewandowsky, Farrell, Jarrold, & Greaves, 2012; von Bastian & Oberauer, 2013), and fluid intelligence (Engle, Tuholski, Laughlin, & Conway, 1999). In short, there exists a rich literature in which the specific cognitive mechanisms underlying working memory in adulthood, and its relationship with other cognitive processes, are keenly debated.

Considerable progress in understanding the cognitive processes of WM has been provided by the analysis of latent factors underlying the wide range of measures of WM that have been developed. Using this individual differences approach, the three‐factor structure has been robustly reproduced across multiple studies and age groups (Alloway, Gathercole, Willis, & Adams, 2004; Kane et al., 2004; Bayliss, Jarrold, Gunn, & Baddeley, 2003; Hornung, Brunner, Reuter, & Martin, 2011), although studies that have drawn on a wider range of assessments indicate that refinements may be needed in the concept of attentional control within the system (Gray et al., 2017). In general, these analyses have favoured the distinction between domain‐specific storage for verbal and visuo‐spatial material linked with an executive or attentional component. These components are already detectable in children from about 5 years of age (Alloway et al., 2004) and their configuration remains broadly stable throughout childhood (Gathercole et al., 2004). Working memory performance, however, improves substantially over childhood (Gathercole et al., 2004; Huizinga et al., 2006; Siegel & Ryan, 1988), with linear increases until adolescence, when adult levels are reached (Gathercole et al., 2004; Luciana, Conklin, Hooper, & Yarger, 2005). It has been widely recognized that the cognitive mechanisms contributing to improvements across different periods may themselves change (Gathercole et al., 2004; Huizinga et al., 2006; Siegel & Ryan, 1988). Developmental improvements in WM may, for example, be driven by increases in storage capacity (Cowan, Ricker, Clark, Hinrichs, & Glass, 2014) and / or attention (Barrouillet, Gavens, Vergauwe, Gaillard, & Camos, 2009; Tam, Jarrold, Baddeley, & Sabatos‐DeVito, 2010). They may also be the consequences of changes in rehearsal strategies (Gathercole, Adams, & Hitch, 1994; Hitch, Halliday, Schaafstal, & Heffernan, 1991), although it is now understood that limitations in the sensitivity of memory span in pre‐school children may obscure the clear signatures of phonologically based rehearsal in older children and adults (Jarrold, 2016; Wang, Logie, & Jarrold, 2016).

### 1.2 Neural correlates of working memory

The developmental period associated with increases in working memory is accompanied by pronounced changes in brain structure. These include decreasing cortical thickness (Sowell, 2004) and increasing myelination of white matter tracts (Dean et al., 2014). Further, functional neuroimaging studies suggest that improvements in working memory are accompanied by some reorganization in brain networks (Houde, Rossi, Lubin, & Joliot, 2010). In adults, a specialized network including bilateral parietal, cingulate, and prefrontal areas has been found to show increased blood oxygenation during working memory tasks (Owen, McMillan, Laird, & Bullmore, 2005; Wager & Smith, 2003). Children show activation in a similar set of regions (Thomason et al., 2009) and also in additional non‐specific areas outside of the core processing network observed in adults (Ciesielski, Lesnik, Savoy, Grant, & Ahlfors, 2006; Vogan, Morgan, Powell, Smith, & Taylor, 2016).

Findings from the more limited research on structural neural correlates of working memory broadly concur with this pattern of change. Frontal and parietal grey matter volume (Mahone, Martin, Kates, Hay, & Horska, 2009; Rossi et al., 2013), and temporal and parietal connections of the corpus callosum (Treble et al., 2013), are significant predictors of a participant's working memory capacity. However, these studies either investigate narrow age ranges or statistically correct for the effect of age. As a result, little is known about how structural brain changes support the development of particular cognitive skills such as working memory. Furthermore, the majority of previous studies have used performance on individual tasks to measure working memory ability (see Poldrack & Yarkoni, 2016, for a detailed discussion). This approach has two key limitations. First, it is widely accepted that multiple underlying components underpin performance (Alloway et al., 2004; Clair‐Thompson & Gathercole, 2006; Conway, Cowan, Bunting, Therriault, & Minkoff, 2002; Oberauer, Süß, Schulze, Wilhelm, & Wittmann, 2000). Second, scores on individual tests also reflect task‐specific components that may be unrelated to WM demands such as proficiency in the stimulus domain from which the stimuli are drawn (Dark & Benbow, 1994). The purpose of the current study was to redress these two gaps in the literature by (i) exploring how structural brain correlates of working memory, in terms of both grey and white matter, differ over developmental time; and (ii) using multiple behavioural assessments alongside factor analysis, to differentiate the neural correlates of robustly determined cognitive components of WM.

## 2 METHODS

**Figure 1:**
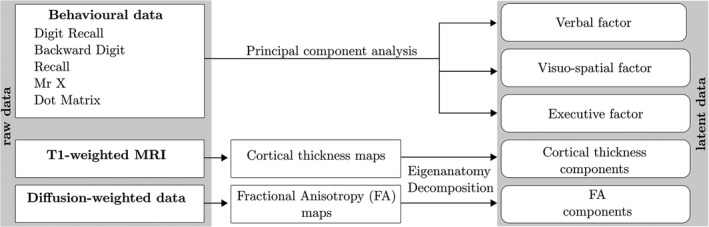
Overview of processing steps from raw to latent data. Raw behavioural data were decomposed with principal component analysis (PCA) to derive factor scores that corresponded to a verbal, visuospatial, and executive factor. Dimensionality reduction was also applied to cortical thickness maps and FA maps derived from T1‐weighted and diffusion‐weighted MRI data to obtain eigenanatomy components

Our analysis approach used data reduction techniques to reduce raw behavioural and neuroimaging measures to underlying statistical components. We then explored how the underlying cognitive factors of the working memory system were associated with structural brain components and the extent to which these relationships were moderated by developmental stage (i.e., age). A schematic summary of this approach can be seen in Figure 1. The computer code used for data processing and statistical analysis is available online (https://github.com/joebathelt/WorkingMemory_and_BrainStructure_Code).

### 2.1 Participants

The data for the current study were taken from two large‐scale studies at the MRC Cognition and Brain Sciences. Both studies employed the same working memory assessments and structural scanning protocols. These two studies had different recruitment criteria but when combined, provide a large sample of children with working memory scores whose distributional properties closely approximated the standardization sample. The first study was the Centre for Attention, Learning, and Memory (CALM) research clinic (*n* = 111, 78 boys, Age [years]: mean = 9.54, std = 2.109, range = 5.58–15.92). At the clinic, children were recruited on the basis of ongoing problems in attention, learning and memory reported by professionals working in schools or specialist children's community services. Exclusion criteria for referrals were a known history of brain injury, significant or severe known problems in vision or hearing that were uncorrected and having a native language other than English. This study was approved by the local NHS research ethics committee (Reference: 13/EE/0157). Written parental consent was obtained and children provided verbal assent. Children attending the clinic completed a cognitive test battery administered over approximately 3 hours. Here, we report data from the working memory measures in this battery.

The second study investigated the neural, cognitive, and environmental markers of risk and resilience in children, and recruited a broad community sample (*n* = 42, 24 boys, Age [years]: mean = 9.95, std = 1.528, range = 7.17–12.42). Children attending mainstream school in the UK with normal or corrected‐to‐normal vision or hearing and no history of brain injury were recruited via local schools and through advertisements in public places (childcare and community centres, libraries). Participating families were invited to the MRC Cognition and Brain Sciences Unit for a 2‐hour assessment that included the working memory battery reported here. Participants received monetary compensation for taking part in the study. This study was approved by the Psychology Research Ethics Committee at the University of Cambridge (Reference: Pre.2015.11). Parents provided written informed consent.

**Figure 2:**
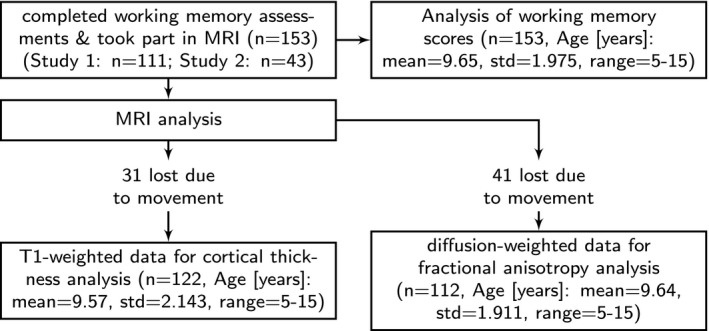
Overview of sample included in the behavioural and neuroimaging analysis

The final sample for behavioural analysis consisted of 153 children between 5.58 and 15.92 years (96 boys, Age [years]: mean = 9.65, std = 1.975, range = 5–15, see Figure 2). Thirty‐one children were excluded from cortical thickness analysis because the T1‐weighted data were not usable due to participant movement (*n* = 122, 67 boys, Age [years]: mean = 9.57, std = 2.143, range = 5–15). Forty‐one children were excluded from the analysis of diffusion‐weighted data due to head movement above 3 mm in the DWI sequence (*n* = 112, 67 boys, Age [years]: mean = 9.64, std = 1.911, range = 5–15). Residual movement estimates were included as a nuisance variable in regression models. As these measures did not influence the results, they were omitted from the reported models.

### 2.2 Working memory assessment

The Digit Recall, Backward Digit Recall, Dot Matrix, and Mr X task of the Automatic Working Memory Assessment (AWMA) (Alloway, 2007; Alloway, Gathercole, Kirkwood, & Elliott, 2008) were administered individually. In Digit Recall, children repeat sequences of single‐digit numbers presented in an audio format. In Backward Digit Recall, children repeat the sequence in backwards order. These tasks were selected to engage verbal short‐term and working memory, respectively. For the Dot Matrix task, the child was shown the position of a red dot for 2 seconds in a series of four by four matrices and had to recall this position by tapping the squares on the computer screen. In the Mr X task, the child was shown two Mr X figures and had to identify whether they were holding the ball in the same or different hands. One Mr X was rotated in each trial. The child then had to recall the location of the ball in Mr X's hand by pointing to one of eight compass points. These tasks were aimed at tapping short‐term and working visuospatial memory.

Standardized scores established that the sample performed at expected levels for their age, that is, mean of 100 and a standard deviation of 15 (Digit Recall: mean = 96.39; std = 16.32; Backward Digit Recall: mean = 94.61, std = 12.671; Dot Matrix: mean = 98.29, std = 15.595; Mr X: mean = 99.32, std = 15.69).

In order to reconstruct the latent variable structure of working memory from the assessment data, principal component analysis was applied. This was carried out using the ‘principal’ function of the psych package v1.5.1 (http://personality-project.org/r) in R v3.1.3 (R Development Core Team, 2008). Varimax rotation was used to create orthogonal factors (Kaiser, 1958). A three‐factor solution provided the best fit with theoretical predictions and explained a large proportion of variance in the assessment scores (92% of the variance in the raw scores). An additional benefit of using the three‐factor solution is that our findings can be readily interpreted alongside, and usefully integrated with, the large behavioural literature on typical and atypical working memory development. Mahalanobis distance was computed to detect outliers in the assessment data, but no data point exceeded the standard cut‐off at 3 degrees of freedom.

### 2.3 MRI data acquisition

Magnetic resonance imaging data were acquired at the MRC Cognition and Brain Sciences Unit, Cambridge UK. All scans were obtained on the Siemens 3 T Tim Trio system (Siemens Healthcare, Erlangen, Germany), using a 32‐channel quadrature head coil. The imaging protocol consisted of two sequences: T1‐weighted MRI and a diffusion‐weighted sequence.

T1‐weighted volume scans were acquired using a whole brain coverage 3D Magnetization Prepared Rapid Acquisition Gradient Echo (MP‐RAGE) sequence acquired using 1 mm isometric image resolution. Echo time was 2.98 ms, and repetition time was 2250 ms.

Diffusion scans were acquired using echo‐planar diffusion‐weighted images with an isotropic set of 60 non‐collinear directions, using a weighting factor of b = 1000s*mm^−2^, interleaved with a T2‐weighted (b = 0) volume. Whole brain coverage was obtained with 60 contiguous axial slices and isometric image resolution of 2 mm. Echo time was 90 ms and repetition time was 8400 ms.

### 2.4 Processing of diffusion‐weighted data

Diffusion imaging makes it possible to quantify the rate of water diffusion in the brain. In the parallel bundles of white matter, diffusion is stronger along the fibre orientation but is attenuated in the perpendicular direction. This can be summarized by the metric of fractional anisotropy (FA), which is a scalar value between 0 and 1 describing the degree of anisotropy of the diffusion at every voxel. Developmental studies show steady increases in FA between childhood and adulthood (Imperati et al., 2011; Muftuler et al., 2012; Westlye et al., 2009), which is likely to reflect increased myelination (Dean et al., 2014).

A number of processing steps are necessary to derive FA maps from diffusion‐weighted volumes. In the current study, diffusion‐weighted MRI scans were converted from the native DICOM to compressed NIfTI‐1 format using the dcm2nii tool (http://www.mccauslandcenter.sc.edu/mricro/mricron/dcm2nii.html). Subsequently, the images were submitted to the DiPy v0.8.0 implementation (Garyfallidis et al., 2014) of a non‐local means de‐noising algorithm (Coupe et al., 2008) to boost the signal‐to‐noise ratio. Next, a brain mask of the b0 image was created using the brain extraction tool (BET) of the FMRIB Software Library (FSL) v5.0.8. Motion and eddy current correction were applied to the masked images using FSL routines. The corrected images were re‐sliced to 1 mm resolution with trilinear interpolation using in‐house software based on NiBabel v2.0.0 functions (http://nipy.org/nibabel/). Finally, fractional anisotropy maps were created based on a diffusion tensor model fitted through the FSL dtifit algorithm (Behrens et al., 2003; Johansen‐Berg et al., 2004).

For comparison across participants, we created a study‐specific FA‐template based on all available images using Advanced Normalization Tools (ANTs) algorithms (Avants et al., 2014; Lawson, Duda, Avants, Wu, & Farah, 2013), which showed the highest accuracy in software comparisons (Klein et al., 2009; Murphy et al., 2011; Tustison et al., 2014). Individual images were transformed to template space using non‐linear registration with symmetric diffeomorphic normalization as implemented in ANTs (Avants, Epstein, Grossman, & Gee, 2008). Next, the images were eroded twice with a 3 mm sphere using FSL maths to remove brain edge artefacts.

### 2.5 Processing of T1‐weighted data

Another measure of brain development that can be derived from neuroimaging data is cortical thickness (Giedd & Rapoport, 2010; Gogtay et al., 2004). Cortical thickness is defined as the distance between the outer edge of cortical grey matter and subcortical white matter (Fischl & Dale, 2000). To obtain thickness measures from anatomical MRI data, T1‐weighted volumes were initially co‐registered with MNI152 space using rigid co‐registration to obtain good initial between‐subject alignment and optimal field of view. Next, all images were visually inspected and images with pronounced motion artefact were removed from further analysis (*n* = 31, 20.25% of the acquired data). The remaining data were submitted to the automatic ANTs cortical thickness pipeline (antsCorticalThickness). Details about the processing pipeline and thickness estimation are described in Tustison et al. (2014) and Das, Avants, Grossman, and Gee (2009). Tissue priors were taken from the OASIS‐TRT‐20 template (http://www.mindboggle.info/data.html#mindboggle-software-data). Subsequently, images in template space were smoothed using a 10 mm full width at half maximum (FWHM) Gaussian kernel and resampled to 2 mm resolution. A thickness mask was created by averaging all images and binarizing the resulting mean image at a threshold of 0.1.

### 2.6 Eigenanatomy decomposition

**Figure 3:**
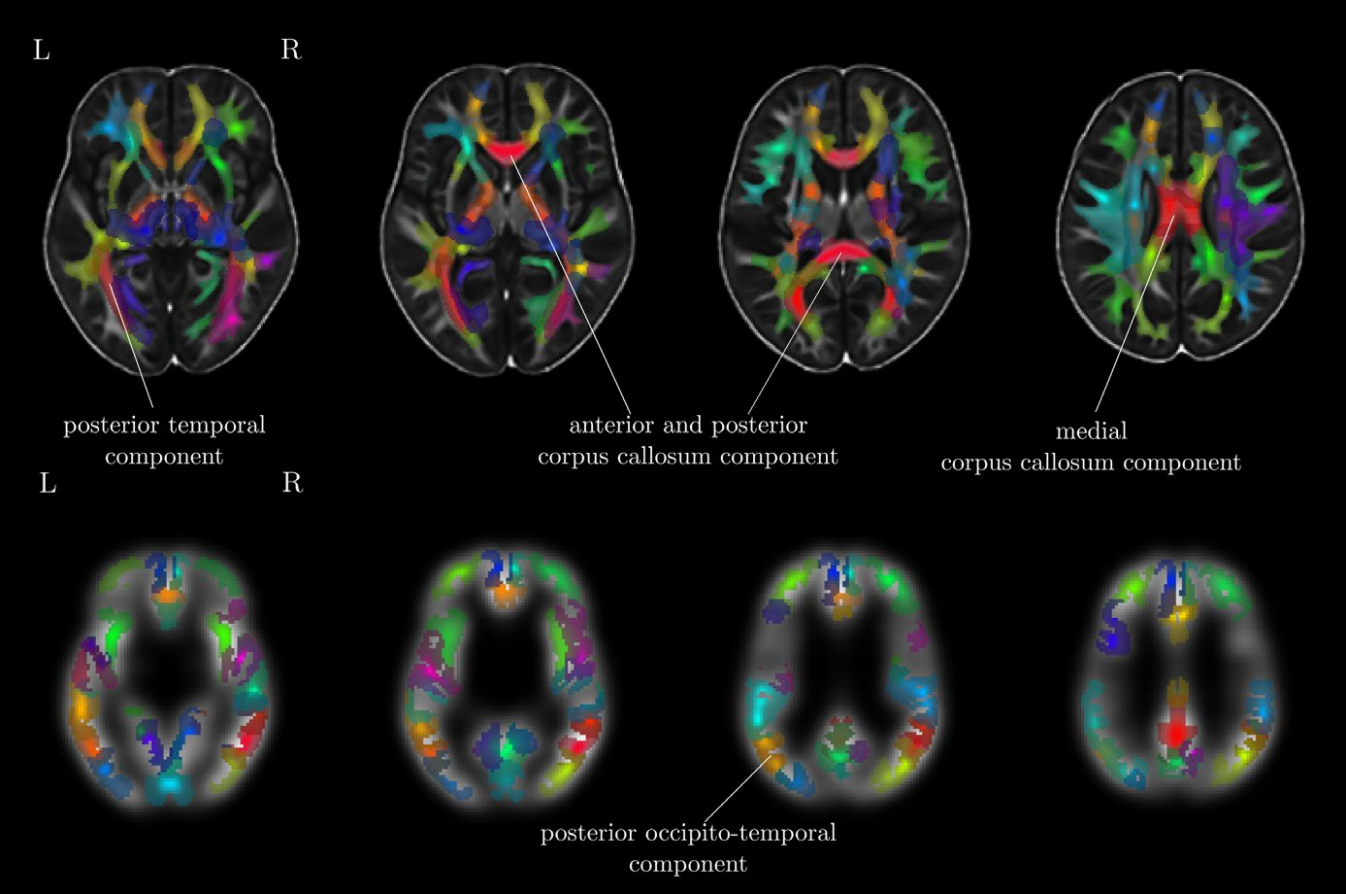
Overview of the eigenanatomy decomposition for FA images (top) and cortical thickness maps (bottom). The 32 components indicated by eigenatomy decomposition are shown on top of the study‐specific FA and cortical thickness template. Cortical thickness images were down‐sampled and smoothed. Labels indicate the components that were found to show interactions with working memory scores and age

Traditional univariate approaches such as voxel‐based morphometry (VBM) fit a statistical model for every voxel in a brain image. The large number of voxels in a typical imaging protocol necessitates correction for a very large number of comparisons (T1‐volumes in the current study contained over 1 million voxels), and this results in a substantial loss of statistical power. However, effects are typically spread over areas that are larger than 1 voxel. Multivariate approaches are better suited to reduce the dimensionality of the data to the information contained in the data themselves before statistical comparisons are applied. Eigenanatomy decomposition is a novel method for data‐driven dimensionality reduction of neuroimaging data that adds sparseness and smoothness constraints for better anatomical interpretability in comparison to standard spatial principal component analysis (Kandel, Wang, Gee, & Avants, 2015). Cortical thickness masks and FA images were processed using the ANTsR v0.3.2 implementation of the eigenanatomy decomposition algorithm (Kandel et al., 2015). Parameters for eigenanatomy decomposition were adopted from published work, that is, decomposition into 32 components with a sparseness of 1/32 with 20 iterations, an L1 penalty with gradient step size 0.5, a smoothing kernel of 1 voxel, and a minimum cluster size of 1000 voxels for each eigenvector. For statistical analysis, the mean value of each brain morphology measure (FA, cortical thickness) within each eigenanatomy component was calculated. See Figure 3 for an illustration of the resulting parcellation.

### 2.7 Statistical analysis

**Figure 4:**
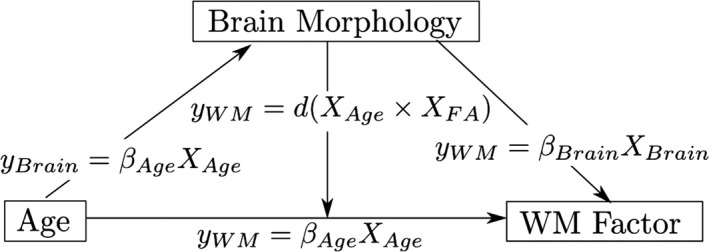
Relationships between age, brain morphology, and working memory factors explored in the current analysis. The relationship between age and working memory factors (verbal, executive, spatial), age and brain morphology measures (FA, cortical thickness), and the interaction effect between age and brain morphology on working memory factors was investigated. All models further contained gender as a regressor of no interest as well as an intercept term and error term. The interaction model also contains terms for age and brain morphology separately. Models for cortical thickness analysis also contained intracranial volume as a regressor of no interest

Our aim was to examine whether and how brain morphology is associated with the components of the working memory system, and the extent to which this relationship is moderated by age. The relationship between these factors was tested in the following set of regression models: (a) age predicting working memory performance, (b) age predicting brain morphology measures, (c) brain morphology predicting working memory; and ultimately (d) the interaction between brain morphology and age predicting working memory (see Figure 4 for an overview of these models). Gender and an intercept term were included as additional regressors in each model. Models for cortical thickness contained intracranial volume as an additional regressor of no interest. Assessment of Cook's distance (Cook, 1977) indicated no particularly influential data points in the regression models. Therefore, all available data points were retained in the analysis. Regression analysis was carried out using the ‘stats’ package v3.1.2 in Rbase. Bonferroni correction was applied to account for multiple comparisons and the adjusted *p*‐values are reported as *p*_corrected_.

## 3 RESULTS

### 3.1 Factor analysis of behavioural data

Principal component analysis (PCA) was applied to the raw scores of the working memory battery to derive the latent variable structure of working memory. Assessment of Mahalanobis distance did not indicate outliers in the cognitive scores (Maximum distance D^2^(4) = 15.542, critical value = 18.47). Correlations between raw scores were moderate to high (range: 0.39 to 0.63). The three‐factor PCA solution explained 92% of the variance in the raw scores. Factor loadings are shown in Table 1.

**Table 1:** Loading of factors based on principal component analysis using varimax rotations of the raw working memory scores. The three‐factor solution explained 93% of the variance. The factor loadings suggested a verbal and spatial storage factor, and an executive factor

	Verbal Factor	Executive Factor	Visuo‐spatial Factor
Digit Recall	0.95	0.15	0.18
Backward Digit Recall	0.55	0.50	0.47
Dot Matrix	0.22	0.27	0.93
Mr. X	0.17	0.94	0.26
Proportion explained	0.35	0.33	0.32
Cumulative proportion	0.35	0.68	1.00

### 3.2 Working memory performance improves with age

**Figure 5:**
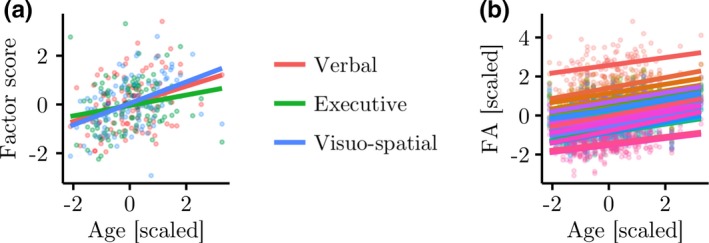
(a) Relationship between age and verbal, executive, and visuospatial factor scores. Linear regression analysis indicated significantly higher scores in older participants for all factors. (b) Relationship between age and FA within eigenanatomy components. Higher FA was significantly related to age in 30 out of 32 eigencomponents (shown)

Linear regression indicated that age was significantly associated with increases in working memory scores (Effects of age including gender as a nuisance regressor: Verbal factor: *F*(2, 150) = 4.538, *p* = .012, *R* ^2^ = 0.057, *R* ^2^_Adjusted_ = 0.044, β_Age_ = 0.010, *t*_Age_(150) = 2.99, *p* = .003; Executive factor: *F*(2, 150) = 6.506, *p* = .002, *R* ^2^ = 0.079, *R* ^2^_Adjusted_ = 0.068, β_Age_ = 0.003, *t*_Age_(150) = 3.09, *p* = .002; Spatial factor: *F*(2, 150) = 16, *p* < .001, *R* ^2^ = 0.176, *R* ^2^_Adjusted_ = 0.165, β_Age_ = 0.018, *t*_Age_(150) = 5.66, *p* < .001; see Figure 5a). Comparison with alternative quadratic and cubic models using the Akaike Information Criterion (AIC) as a measure of parsimony (Akaike, 1974) suggested that a linear model provided the best account for the relationship between age and factor scores in the current data (Verbal factor: AIC_linear_ = 433.12, AIC_quadratic_ = 432.21, AIC_cubic_ = 427.54; Executive factor: AIC_linear_ = 428.46, AIC_quadratic_ = 429.52, AIC_cubic_ = 431.51; Spatial factor: AIC_linear_ = 408.91, AIC_quadratic_ = 409.94, AIC_cubic_ = 411.61).

### 3.3 FA increases with age

In order to assess the relationship between each measure of brain morphology and participant age, a linear regression analysis was carried out (FA: y_FA_ = β_Age_X_Age_ + β_Gender_X_Gender_ + β_Intercept_ + ε; Cortical thickness: y_Thickness_ = β_Age_X_Age_ + β_Gender_X_Gender_ + β_ICV_X_ICV_ + β_Intercept_ + ε). For FA, the results indicated a significant effect of age in 30 of the 32 components after Bonferroni correction for multiple comparisons. The effect was marginal for the remaining two components after correction for multiple comparisons (*p* < .051). The slopes were positive for all components (β_Age_: mean = 0.22, *SD* = 0.03, Range = 0.16–0.29, based on *z*‐scores; see Figure 5b), indicating that FA increased with age for all eigenanatomy components. For cortical thickness, the results indicated no significant relationship with age (β_Age_: mean = 0.05, *SE* = 0.01, Range = −0.08–0.18, based on *z*‐scores).

### 3.4 FA predicts differences in executive scores

Next, the relationship between brain morphology and factor scores was assessed (FA: y_Factor_ = β_FA_X_FA_ + β_Gender_X_Gender_ + β_Intercept_ + ε; Cortical thickness: y_Factor_ = β_Thickness_X_Thickness_ + β_ICV_X_ICV_ + β_Gender_X_Gender_ + β_Intercept_ + ε). There was no significant effect of FA in any eigenanatomy component for the verbal and visuospatial storage factor after correction for multiple comparisons. There were significant effects of FA in 16 eigenanatomy components for the executive factor (see Table 2). For cortical thickness, the results indicated no significant effect of cortical thickness within any of the 32 eigenanatomy components on scores for any of the working memory factors (corrected‐*p* > .05). In summary, FA predicted working memory capacity associated with the executive factor, while cortical thickness was not significantly associated with any working memory constructs.

**Table 2:** FA within eigenanatomy components that showed significant linear relationships with executive function scores. The coordinates refer to the position of the ROI centroid in MNI152 space

		Volume	x	y	z	β	tstat	*p*	*p*_corrected_
Comp 2	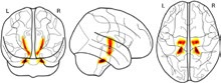	46439	90.71	83.23	84.62	0.47	3.09	0.003	0.01
Comp 3	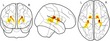	41374	89.78	101.47	59.97	0.48	2.77	0.007	0.03
Comp 4	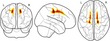	46585	91.01	107.09	85.48	0.49	2.74	0.007	0.031
Comp 6	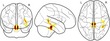	32929	106.63	119.04	96.72	0.45	2.64	0.009	0.041
Comp 7	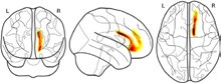	33840	78.00	92.32	57.81	0.48	2.68	0.009	0.035
Comp 8	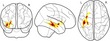	23804	74.90	152.59	82.53	0.46	2.88	0.005	0.03
Comp 9	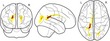	24972	125.41	106.25	65.33	0.51	3.15	0.002	0.008
Comp 10	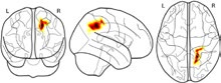	36812	97.46	75.41	92.97	0.5	3.24	0.002	0.007
Comp 13	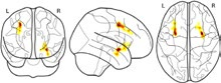	27930	98.03	82.84	99.30	0.49	2.91	0.004	0.019
Comp 16	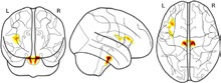	20309	76.13	101.56	101.69	0.41	2.71	0.008	0.04
Comp 20	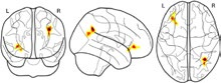	20608	110.17	112.02	100.88	0.46	2.79	0.006	0.033
Comp 22	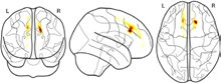	18040	86.64	98.73	71.46	0.49	2.9	0.005	0.017
Comp 24	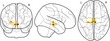	21049	64.55	115.31	75.93	0.51	2.73	0.007	0.033
Comp 25	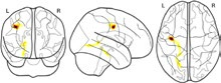	15867	94.10	104.76	67.27	0.53	3.11	0.002	0.01
Comp 27	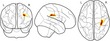	19398	66.55	116.81	99.49	0.51	2.99	0.003	0.02
Comp 28	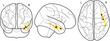	13506	54.45	102.02	102.15	0.46	2.65	0.009	0.036

**Figure 6:**
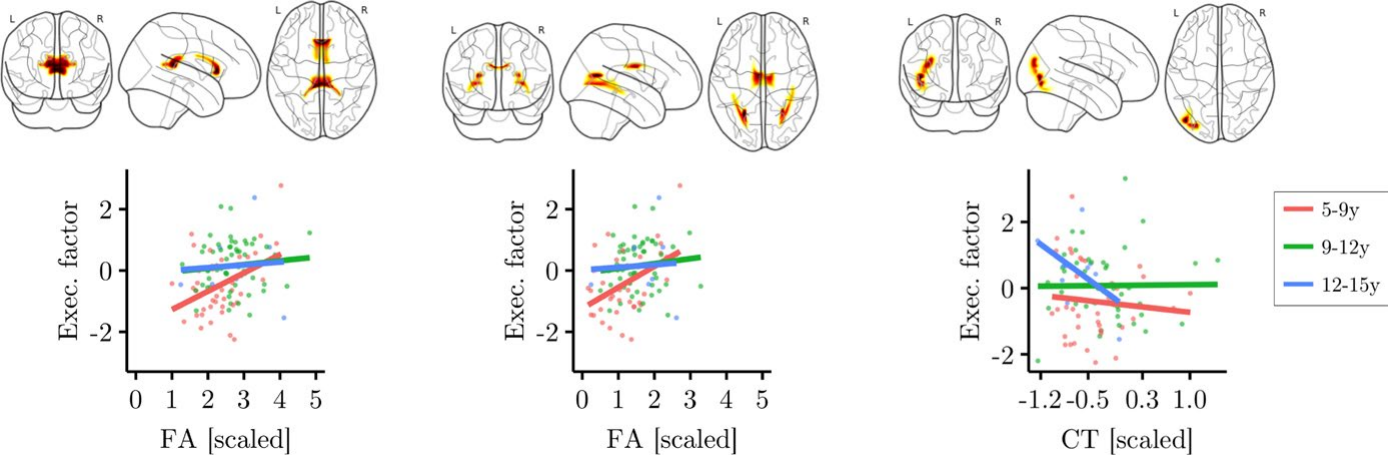
Interaction effect of age and measures of brain morphology (FA, cortical thickness) on executive factor scores. Age was split into three groups for better visualization of the results, but was treated as a continuous variable in the main analysis. Glass brain maps represent the topography of the components in MNI space. Regression analysis indicated significant interactions in two FA components (anterior and posterior corpus callosum, medial corpus callosum and bilateral posterior temporal white matter). FA in these components was more predictive of executive scores in younger children. For cortical thickness, one component in the left occipitotemporal cortex showed a significant interaction effect with age. In this component higher cortical thickness was more predictive of lower executive function scores in older children. One data point with extreme cortical thickness values has been removed for this illustration. Removing this data point did not influence the results of the main analysis, but made this figure more difficult to interpret

Finally, the extent to which the relationship between brain morphology and components of the working memory system is moderated by age was investigated. The regression model for this analysis controlled the linear contributions of age, gender, and FA within the eigenanatomy component, and also contained a term for the interaction between FA and age which was the main focus of this analysis (y_Factor_ = β_Age_X_Age_ + β_FA_X_FA_ + d(X_Age_ × X_FA_) + β_Gender_X_Gender_ + β_Intercept_ + ε). The results indicated a significant effect of the interaction between age and FA on the executive factor in two eigenanatomy components (Corpus callosum component: β = −0.337, *t*(5) = −3.35, *p* = .001, *p*_corrected_ = .036; Occipitotemporal white matter component: β = −0.368, *t*(5) = −3.32, *p* = .001, *p*_corrected_ = .039; see Figure 6).

For cortical thickness, intracranial volume was included as an additional regressor of no interest (y_Factor_ = β_Age_X_Age_ + β_Thickness_X_Thickness_ + d(X_Age_ × X_Thickness_) + β_Gender_X_Gender_ + β_ICV_X_ICV_ + β_Intercept_ + ε). The results of the regression analysis indicated a significant interaction between age and cortical thickness for one eigenanatomy component (left temporal thickness component: β = 0.56, *t*(5) = −0.91, *p* = .002, *p*_corrected_ = .049).

## 4 DISCUSSION

The aim of the current study was to explore how structural brain correlates of working memory capacity differ with age. The neural structures associated with the executive component of the working memory system were shown not to be invariant across age, but to interact with it. Specifically, the corpus callosum and bilateral posterior temporal white matter, and cortical thickness in the left occipitotemporal cortex made differential contributions to the executive component of working memory according to age.

Performance on four tasks was used to assess the latent structure of working memory. Distinct factors were identified for verbal and visuo‐spatial storage with an additional factor contributing to tasks with a higher executive demand load regardless of domain (see also Alloway et al., 2004; Bayliss et al., 2003; Kane et al., 2007). Factor scores were linearly related to age for all factors, replicating previous studies that indicate linear increases in short‐term and working memory capacity throughout childhood and adolescence (Conklin, Luciana, Hooper, & Yarger, 2007; Gathercole et al., 2004; Swanson, 1999).

### 4.1 White matter organization but not cortical thickness show differences with age

Next, the aspects of neurophysiology that show the greatest degrees of age‐related differences were investigated. Of particular interest was the anatomy of white matter. White matter changes are thought to be a key process in postnatal brain development, which continues throughout childhood and adolescence into early adulthood (Barnea‐Goraly, 2005; Muftuler et al., 2012; Qiu, Tan, Zhou, & Khong, 2008; Tau & Peterson, 2010). In particular, the myelination of axons is thought to be a critical mechanism of brain development in this age range (Miller et al., 2012). Differences in microstructural properties, namely FA, as measured by diffusion MRI are directly related to myelination and have been linked to cognitive development (Clayden et al., 2012; Mabbott, Noseworthy, Bouffet, Laughlin, & Rockel, 2006). In the current study, FA was also significantly related to age.

In contrast, cortical thickness was not related to age. This was unexpected as multiple studies have reported decreasing cortical thickness with age (Sowell, 2004; Tamnes et al., 2009; Tamnes et al., 2010; Wierenga, Langen, Oranje, & Durston, 2014). However, these studies included participants from early childhood to adulthood (Tamnes et al., 2009, Sowell et al., 2006, Wierenga et al., 2014), or mapped changes longitudinally over a shorter period (Shaw et al., 2006; Sowell, 2004). Our narrower age range could account for the absence of age‐related differences in cortical thickness in the current study. Even so, our data indicate that FA is a more sensitive indicator of brain development in the 6‐ to 16‐year age range, and that neural differences across this age span are largely mediated by the maturation of structural connections and integration within brain systems.

### 4.2 Brain morphology and age interact in the development of the executive component of working memory

Many studies indicate that neural correlates of cognitive development show a shift from using general brain systems in younger children to an adult‐like recruitment of specialized networks of regions in older participants (Johnson, 2011). This developmental tendency has been demonstrated for the processing of both faces (Kadosh, Johnson, Henson, Dick, & Blakemore, 2013; Kadosh, Kadosh, Dick, & Johnson, 2010) and language (Weiss‐Croft & Baldeweg, 2015). Functional neuroimaging studies indicate that this developmental progression may also apply to working memory. Children show higher blood oxygenation in additional regions beyond the core working memory areas found in adults (Ciesielski et al., 2006; Crone, Wendelken, Donohue, van Leijenhorst, & Bunge, 2006; Scherf, Sweeney, & Luna, 2006; Vogan et al., 2016). However, differences in the contribution of brain structure with development have not been investigated so far.

The current study indicates that younger children's working memory capacity is more closely associated with the microstructural integrity of white matter components than is the capacity of older children and adolescents. In contrast, higher focal cortical thickness in areas associated with working memory is associated with lower levels of performance in older but not in younger children. In short, the executive aspects of working memory are supported by different brain systems across this age range. The greater importance of large white matter connections in younger children suggests that younger children are relying on a more distributed system. In contrast, the greater importance of cortical thickness in the left posterior temporal lobe demonstrates the importance of this local processing in later stages of working memory development.

There exists a relatively large literature discussing the potential functional role of the particular structures that were implicated in our findings. To summarize briefly: it has been hypothesized that interhemispheric connections of the corpus callosum provide inhibition between functionally homologous areas in the left and right hemisphere (Gazzaniga, 2000). Differences in corpus callosum anatomy such as reduced volume or reduced microstructural integrity are related to lower lateralization of function in typical participants and patient groups (Hinkley et al., 2016; Just, Cherkassky, Keller, Kana, & Minshew, 2007; Persson et al., 2006). In turn, lower lateralization is associated with lower performance on cognitive tasks, including executive function tasks (Hinkley et al., 2012; Just et al., 2007; Nagel, Herting, Maxwell, Bruno, & Fair, 2013). Similarly, posterior temporal white matter may provide connections for integration between specialized regions of temporal lobe for verbal and visuospatial working memory with the posterior parietal executive attention network. Posterior temporal white matter has also been shown to relate to working memory performance in typical adults (Burzynska et al., 2011; Golestani et al., 2014) and in lesion studies (Finke, Bublak, & Zihl, 2006; Palacios et al., 2012).

With structural data alone, we are left to speculate as to the functional role of these connections and their changing contribution to working memory capacity across development. But our findings make a more fundamental point – despite the factor structure of working memory being stable across childhood (Alloway et al., 2004; Gathercole et al., 2004; Luciana et al., 2005), the neural systems associated with it change. Impairments of working memory are a consistent feature of numerous neurodevelopmental disorders (Archibald & Gathercole, 2006; Gathercole & Baddeley, 1989; Holmes et al., 2014; Martinussen et al., 2005; Montgomery, 2000; Rotzer et al., 2009; Smith‐Spark & Fisk, 2007; Szucs et al., 2013; Weismer et al., 1999), in addition to being an important constraint on learning within the typically developing population (Cain et al., 2004; Clair‐Thompson & Gathercole, 2006; Cowan, 2013; Dumontheil & Klingberg, 2011; Gathercole, Tiffany, Briscoe, & Thorn, 2005; Gathercole et al., 2003). Understanding the nature of these impairments, and providing a plausible neuropsychological account for them, will require a developmentally informed model of brain–behaviour relationships. Differences in capacity will likely be underpinned by a different combination of neural systems, depending upon the age of the child.

## 5 CONCLUSION

The current study investigated whether the relationship between individual differences in brain structure and working memory performance varies with age. There was clear evidence of differences in the neural underpinnings of the executive component of working memory, with a shift from a higher contribution of callosal and temporal white matter in younger children to a greater dependence on left temporal cortex in older children. The current study can be characterized as a developmental progression from an early distributed system supported by long‐range connections to later reliance on specialized local circuitry.
